# Nonlinear modeling of magnetic materials for circuit simulations

**DOI:** 10.1038/s41598-023-44187-3

**Published:** 2023-10-11

**Authors:** Kamil Kutorasiński, Jarosław Pawłowski, Paweł Leszczyński, Marcin Szewczyk

**Affiliations:** 1 Department of Condensed Matter Physics, Faculty of Physics and Applied Computer Science, AGH University of Krakow, Reymonta 19 St., 30-059 Kraków, Poland; 2 Institute of Theoretical Physics, Wroclaw University of Science and Technology, Wyb. Wyspiańskiego 27 St., 50-370 Wrocław, Poland; 3 Division of Power Apparatus, Protection and Control, Faculty of Electrical Engineering, Electrical Power Engineering Institute, Warsaw University of Technology, Koszykowa 75 St., 00-662 Warszawa, Poland

**Keywords:** Energy infrastructure, Materials for devices

## Abstract

Magnetic materials in the form of magnetic rings are widely used in power engineering products. In many cases, they operate in high frequency and in nonlinear conditions, e.g., as damping elements in electrical power substations equipped with Gas-Insulated Switchgear (GIS) where they provide suppression of Very Fast Transient Overvoltages (VFTOs). To model phenomena in GIS with magnetic rings it is required to have realistic time-dependent models of magnetic materials operating in a wide frequency range and nonlinear conditions. Nowadays, this has become even more relevant due to the actual trend in the industry to create digital twins of physical devices. Models composed of high-precise discrete lumped nonlinear elements are in demand in circuit simulators like SPICE. This work introduces a method based on classical algorithms that find equivalent lumped models of magnetic cores based on frequency-dependent measurements of impedance under DC-bias current. The model is specifically designed to have smooth behavior in the current domain and thanks to that to improve numerical stability in the time domain simulations.

## Introduction

### Problem definition and goal

Magnetic rings play an important role in the electrical power industry by addressing two main challenges: mitigating Electromagnetic Interference (EMI) and suppressing overvoltages in electronics and electrical power devices^1^. Magnetic rings installed in power products operate under various electrical conditions, which can be divided into two categories. The first category involves systems in which the rings operate within the linear range, where the magnetic fields do not cause the magnetic material to saturate. Commonly, this includes the widespread use of chokes in low-current circuits (low-power electronics). The second group involves systems operating in the nonlinear range. These systems involve high currents, such as in Gas-Insulated Switchgear (GIS)^2,3^, where even a single-turn arrangement can cause saturation of the magnetic material. More examples include rings installed onto the shaft of the motor to mitigate bearing currents^4^ or for the mitigation of high frequency transients in wind turbine transformers^5^.

To optimize the design of power products that contain magnetic material in a form of ring there is a need for the model that operate in both linear and nonlinear conditions. Such a model is typically employed in circuit modeling using dedicated software for circuit simulations (e.g., SPICE, EMTP-ATP). A proper model of a magnetic ring enables the selection of a material with appropriate material characteristics (permeability, saturation, and the dependence of these parameters on frequency and magnetic field) in design process of power devices. It also allows for the selection of the geometry and the required amount of material, which affects not only the cost of the ring itself but also the cost and reliability of the device in which it is installed. Therefore, computationally efficient and accurate methods for creating models of magnetic rings are necessary This days this becomes even more relevant because of the desire to create digital twins of power devices^6,7^.

This study explores the effectiveness of classical, non-AI-based algorithms in developing a time-dependent model for magnetic rings that operate across a wide frequency range and under nonlinear conditions^8,9^. The model relies on impedance measurements^10^ that are frequency-dependent and obtained under a DC-bias current^11^. These measurements offer comprehensive insights into the behavior of the magnetic ring when subjected to a combination of a small harmonic forcing and the DC component of the current. Such a scenario occurs in Gas Insulated Switchgear (GIS), where high-frequency currents caused by Very Fast Transients (VFTs) are superimposed on a 50 Hz current^12^. The magnetic material employed in this study is a noncrystalline ring^13^, operating in a single-wire arrangement; however, the modeling methods presented in this research are versatile and are not limited to this particular case.

### Magnetic material modeling

From the perspective of magnetic material modeling, in the case where the magnetic material is in the form of a thin ring, two important dimensional parameters are considered: $A_\mathrm{fe}$, which represents the magnetic material cross-section, and $l_\mathrm{m}$, denoting the magnetic path length. These parameters are crucial for converting impedance data into material-related properties, specifically relative permeability $\mu _\mathrm{r}(f)$, using the following formula^14^:

1 $$\begin{aligned} \mu '&= \mu _\mathrm{r}= L_\mathrm{s} \frac{l_\mathrm{m}}{\mu _0 \ A_\mathrm{fe}},\end{aligned}$$

2 $$\begin{aligned} \mu ''&= R_\mathrm{s} \frac{1}{\omega } \frac{l_\mathrm{m}}{\mu _0 \ A_\mathrm{fe}}, \end{aligned}$$

where the material properties can be described by a complex permeability $\mu = \mu ' - j\mu ''$, which, in general, depends on both the frequency *f* and the magnetic field *H*, denoted as $\mu = \mu (f,H)$. In the case of a magnetic ring with a single wire arrangement, the magnetic field can be represented as $H = i/l_\mathrm{m}$. Therefore, modeling the magnetic material properties $\mu (f,H)$ is equivalent to modeling the complex impedance $Z(f,i) = R_\mathrm{s}(f,i) + j\omega L_\mathrm{s}(f,i)$.

It’s important to note that this formula is a simplified approximation and assumes a uniform magnetic field distribution within the ring. In practice, the magnetic behavior of real materials can be more complex, in particular, the parameters $A_\mathrm{fe}$ and $l_\mathrm{m}$ may depend on *H* and *f*. Thus, the material parameters $\mu _\mathrm{r}$ and $\mu ''$ must be treated as effective parameters for the entire magnetic component.

### Nonlinear lumped series $\textrm{LR}$ model

**Figure 1:**
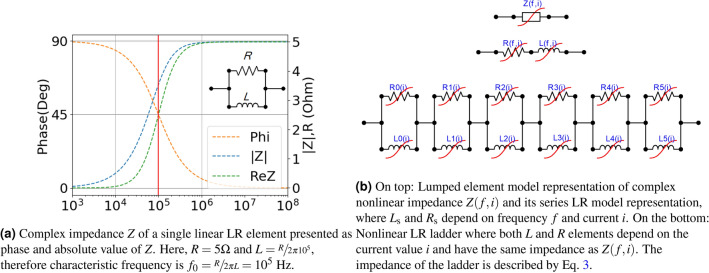
Graphical representation of lumped element model of magnetic ring.

The objective is to develop a time-domain model of the non-linear impedance, denoted as *Z*(*f*, *i*), in the harmonic approximation, which depends on both frequency and current. To achieve this, a circuit model utilizing lumped elements was selected as the most versatile choice, composed of lumped inductors (*L*) and resistors (*R*) connected in a series $\textrm{LR}$ ladder configuration, known as Foster synthesis, extended to $\textrm{LR}$ case^15^. It should be noted, that this approach has limitations, as the impedance of such a system increases with frequency and has a phase angle ranging between $0^{\circ }$ and $90^{\circ }$. In this regard, a single $\textrm{LR}$ element meets these requirements, as depicted in Fig. 1. Consequently, an $\textrm{LR}$ ladder, which is a combination of multiple single $\textrm{LR}$ impedances, will exhibit rising impedance and a phase angle between $0^{\circ }$ and $90^{\circ }$. It is important to recognize that a device modeled using $\textrm{LR}$ elements cannot possess capacitance properties. Magnetic rings in a one-wire arrangement satisfy this assumption.

The commonly used procedure for s-domain rational function fitting is Vector Fitting (VF)^16–19^. In the VF, the poles of the rational function are enforced to be stable, real, and positive as needed here. Nonetheless, the foremost drawback of the VF remains in its inability to deal with sets of Z(s), thus, its limited capability to attain a nonlinear circuit model. In other words, the VF can solely match a polynomial to a single Z(s). However, we have managed to correlate Z(s) fits for various currents, ensuring smooth transitions in resultant parameters, a prerequisite for stable simulations. Therefore, in this work, we seek a more specialized method to assumptions.

Equation Eq. (3) shows the equivalent impedance of the ladder $\textrm{LR}$ where $L_\mathrm{s}$ and $R_\mathrm{s}$ denote, respectively, the inductance and resistance in the series equivalent model, and $s=j\omega$. A graphical representation of the model is shown in Fig. 1. The $f_k$ denotes *k*-th element of characteristic frequency.

3 $$\begin{aligned} Z(s,i)=R_\mathrm{s}(s,i)+sL_\mathrm{s}(s,i)=\sum _{k} \frac{sR_k(i)}{s+ R_k(i)/L_k(i)} = \sum _{k}\frac{sL_k(i)}{1+ s/s_k} , \ \ \ s_k=2\pi f_k = R_k(i)/L_k(i). \end{aligned}$$

A critical aspect of such a system is the requirement for numerical stability during simulation, such as in the SPICE or EMTP circuit simulators^20^. This necessitates that the functions representing the current relationships in the individual $\textrm{RL}$ elements exhibit smooth behavior with respect to current.

An essential consideration is selecting the number of $\textrm{LR}$ elements in the ladder structure. In this publication, the number of elements is selected as 6, which is based on the frequency range of the measurement data (as shown in the subsequent section) and the authors’ expertise. A lower number of elements results in increased total impedance matching error, while a higher number can lead to challenges in maintaining continuous and smooth functions of *L*(*i*) and *R*(*i*) within each ladder element. Consequently, an excessive number of elements can cause numerical issues during simulations.

Another restriction imposed by the authors is that the the inductance and resistance of individual L and R elements values decreases as a function of current. Although this assumption may not be universally applicable, it is adopted here due to the measurement data conforming to this condition.

## Measurement data

The nonlinear complex impedance model *Z*(*f*, *i*) is established based on measurement data. The data were obtained for the noncrystalline material in the form of a ring^21^ with an outer diameter of c.a. 20 cm ($l_\mathrm{m}=59$ cm, $A_\mathrm{fe}=2.78$ cm$^2$). The technique described in^11,22^ was employed, utilizing an impedance analyzer^23^, to capture the impedance data for various DC-bias currents.

**Figure 2:**
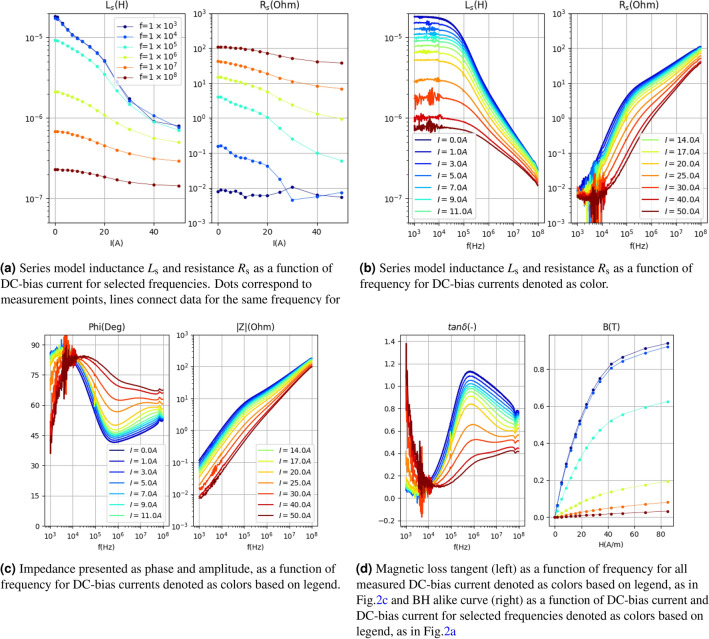
Complex impedance $Z(f,I_{DC})$ of magnetic ring measured with the use of impedance analyzer for various DC-bias currents, presented in four different ways.

The measurement data utilized in this publication consists of complex impedance as a function of frequency for 14 different DC-bias currents, denoted as $Z(f,I_\mathrm{DC})$. The data is presented in Fig. 2. In the frequency domain, the data were collected over a range of six decades from $10^3$ to $10^8$ Hz, with a total of 400 data points for each DC-current value. However, for modeling purposes, only 40 points were selected to represent the impedance characteristics. The DC-bias current values range from zero up to a level that is expected to fully saturate the magnetic material. In this study, the maximum current used was 50 A, which resulted in almost complete saturation of the material, as illustrated in Fig. 2d. Intermediate values of the DC-bias current were manually selected to accurately capture the gradual changes in the impedance.

The data exhibit the following features that are relevant for modeling:

- The measurement data are characterized by a decrease in inductance as a function of current and frequency over their entire range.
- At low frequencies, the impedance has small values, which manifests itself as an increase in the noise visible for $L_\mathrm{s}$ and $R_\mathrm{s}$ caused by the limit of the analyser.
- At low frequencies, $R_\mathrm{s}$ tends towards a certain lower limit, which corresponds to the ohmic resistance of the connection, and not the impedance of the ring itself, which, according to physics, tends towards zero for the frequency tending towards zero. The value of the lead resistance can be estimated to be $8\times 10^{-3} \ \Omega$.
- The circuit does not aim for full saturation (see Fig. 2d), which may be due to the maximum current being too low. However, this does not affect the modeling method itself.

Figure 2d shows a BH curve from data collected from the impedance analyzer. It is worth noting that such a curve is not a typical BH curve but only an approximation. An actual BH curve requires measurements of *Z*(*f*, *i*) for any current *i*. Here, however, low-current measurements are used with an added DC-bias current *Z*(*f*, *i*), where $i=i_{AC}+i_{DC}$, where $i_{AC}$ is a small single harmonic current (a few mA) derived by the analyzer, while $i_{DC}$ is a large value constant current. In this publication, to simplify the notation, $Z(f,i)\approx Z(f,i_{DC})$ is used. The curve shown in Fig. 4 is calculated using the formula:

4 $$\begin{aligned} B(H)=B(i/l_\mathrm{m})=\frac{1}{\mu _0 \ A_\mathrm{fe}}\int _i L(i)di, \end{aligned}$$

where $i=i_{DC}$. This approach allows an approximation of the magnetization curve, which is no more accurate at high frequencies. Additionally, due to the skin effect, the factor $A_\mathrm{fe}$ is no longer constant but decreases as a function of frequency, which is not taken into account here. The accuracy of the above approximation is not discussed in this work.

## Model element parameter fitting

### Optimisation method

The objective is to find all $L_k$ and $R_k$ values for all *k* ladders and all *i* currents. In this work, the maximum *k* number of ladder elements was chosen as 6 based on the frequency range of the measurement data (6 decades) and the experience of the algorithm author. Additionally, to reproduce the correct behavior of the model as a function of current, the parameters $L_k$ and $R_k$ are expected to be a continuous and smooth function of the current. In this publication, due to a sufficiently large number of impedance measurements for different currents $i_\mathrm{DC}$, it was decided to fit a model, i.e., $L_k(i)$ and $R_k(i)$, for each current $i_{DC}$. However, other options are possible, e.g., finding a model for a larger number of points *i* based on the measured data obtained by interpolation in the $i_{DC}$ domain, or finding a model for measured currents $i_{DC}$, even if they are in small numbers, and then interpolating the model itself.

The search for optimal parameters $L_k$ and $R_k$ is performed by minimizing the function:

5 $$\begin{aligned} \min |Z(s,i)-Z_\mathrm{true}(s,i)| = \min \sqrt{\left( Z(s,i)-Z_\mathrm{true}(s,i)\right) ^2}, \end{aligned}$$

where *Z*(*s*, *i*) is expressed by Eq. (3), and $Z_\mathrm{true}(s,i) = Z_\mathrm{true}(s,i_\mathrm{DC})= R_\mathrm{s}( s,i_\mathrm{DC}) + sL_\mathrm{s}(s,i_\mathrm{DC})$ represents the measurement data. In this publication, the optimization of parameters was performed using the method implemented in SciPy library^24^ for solving nonlinear least-squares problems with variable bounds^25^.

The optimization process involves adjusting the values of $L_k$ and $R_k$ for each individual ladder element *k*, resulting in the variation of characteristic frequency $f_k = R_k/(2\pi L_k)$. However, during the iterative optimization process, there is a risk that the calculated value of $f_k$ may deviate significantly from the range of the measured data. This can lead to numerical instability in the iteration process and result in non-optimal solutions. Specifically, if $f_k$ falls significantly below the range of the measured data, the corresponding ladder element may introduce only a purely resistive factor. Conversely, if $f_k$ exceeds the range of the measured data, the ladder element may introduce a highly unstable inductance (see Fig. 1). The optimal solution is achieved when the values of $f_k$ lie within or in close proximity to the range of the measured data. To prevent uncontrolled changes in $f_k$ during the numerical optimization process and simplify and expedite the search for optimal values, a fixed (rigid) grid of characteristic frequencies $f_k$ was employed. This approach ensures stability during optimization and efficiently identifies optimal parameter values ($L_k$ or $R_k$, depending on the approach). Further details will be provided in the subsequent section.

### Method details

The optimization process involves independent minimization of the function defined in Eq. (5) for each current *i*. A fixed grid of six characteristic points $f_k$ was used, with the selection method discussed in the subsequent section. The fixed values of frequencies $f_k$ determine the relationship between $L_k$ and $R_k$, thereby simplifying the optimization process to focus on a single parameter. For numerical simplicity, optimizing $L_k$ is a better choice than the $R_k$. In consequence, this problem can be reduced to a linear equation, which can be expressed in a matrix form as:

6 $$\begin{aligned} \min |Z-Z_\mathrm{true}|\Rightarrow \min |[A]_{ik}.[x]_k-[Z]_i|, \end{aligned}$$

where:

- *k* index denotes the *LR* element, here $k=0,1,\ldots ,5$,
- *i* index represents the index of the frequency grid at which the model function and measurement data functions, *Z* and $Z_{true}$, are compared, here, $i=0,1,\ldots ,29$,
- $[x]_k =x_k = L_k$ denotes the unknown vector being sought, here length of this vector is 6,
- $[A]_{ik}=A_{ik}= \frac{s_k}{(1 + s_k/\omega _i )}$ represents the matrix of constants, here the size of this matrix is $30\times 6$,
- $\omega _i=2\pi f_i$ is a constant associated with the frequency grid at which the model function and measurement data are compared, here length of this vector is 30,
- $s_k = j f_k= j R_k/L_k$ is a constant associated with the characteristic frequencies of the ladder elements, arbitrarily chosen. See the next section for details, here length of this vector is 6, $j=\sqrt{-1}$.

The optimal solution, i.e., $x_k$, was obtained by using a nonlinear least squares method^25^ with bounds on the variables and with the use of the Trust Region Reflective algorithm (TFS) and a 3-point finite difference scheme for numerical estimation of the Jacobian matrix derivatives.

The calculations were performed sequentially, starting from the smallest currents to the largest. The optimization procedure requires selecting an initial vector, denoted as $x_k^{\textrm{init}}$, which is arbitrarily chosen for the first current. In this case, the vector values range from 0 to $L_\mathrm{max}$, logarithmically distributed. Here, $L_\mathrm{max}$ represents the maximum inductance observed in the measurements. For each subsequent current, the initial vector $x_k^{\textrm{init}}$ is set as the vector $x_k$ obtained from the previous current. This approach introduces correlations between consecutive currents, resulting in smooth changes in $x_k$ for every calculation. Consequently, both $L_k(i)$ and $R_k(i)$ exhibit smooth variations with respect to the current, which is facilitated by the constant rigid of $f_k$.

The optimization method employs constraints on the vector $x_k$, limiting the solutions from below 0 and above by the value of $x_k$ from the previous solution (solution for a lower current). This enforces additional correlations between solutions for different currents and ensures the physical property of decreasing inductance over time as the ring enters saturation. The solutions for individual currents are sequentially sought, starting from small currents and progressing to larger ones, although reverse searching is also possible.

Both features of the algorithm, starting the search from the previous current and limiting the next solution to be lower than the previous one, ensure the correlation between the results of searching for $L_k$ for different current. Without these constraints, $L_k$ would be independently computated for each current and would not provide the smooth continuity of the $L_k(i)$ variation as a function of current.

The grid of points $f_i$ should be 2–3 times larger than the number of *LR* ladder elements in the system and should consist of at least several points per decade. Additionally, if the measurement data exhibit noise, a higher number of points is recommended. In this work, a 30-points grid of $f_i$ was selected, beyond which the computational accuracy did not significantly improve. The parameter search for $L_k$ and $R_k$ for all currents takes approximately a few seconds on a standard personal computer.

In practice, it is feasible to optimize functions other than Eq. (5), as demonstrated in the results section, where the relative error minimization function $\min |Z/Z_\mathrm{true}-1|$ is used instead of the absolute error. This modification does not alter the procedure but improves accuracy for data that vary by several orders of magnitude, as here.

The accuracy of the fitting process critically depends on the selection of the grid points $s_k = 2\pi j f_k$. The method for determining the optimal distribution of these points is presented in the subsequent section.

### Characteristic frequency distribution

**Figure 3:**
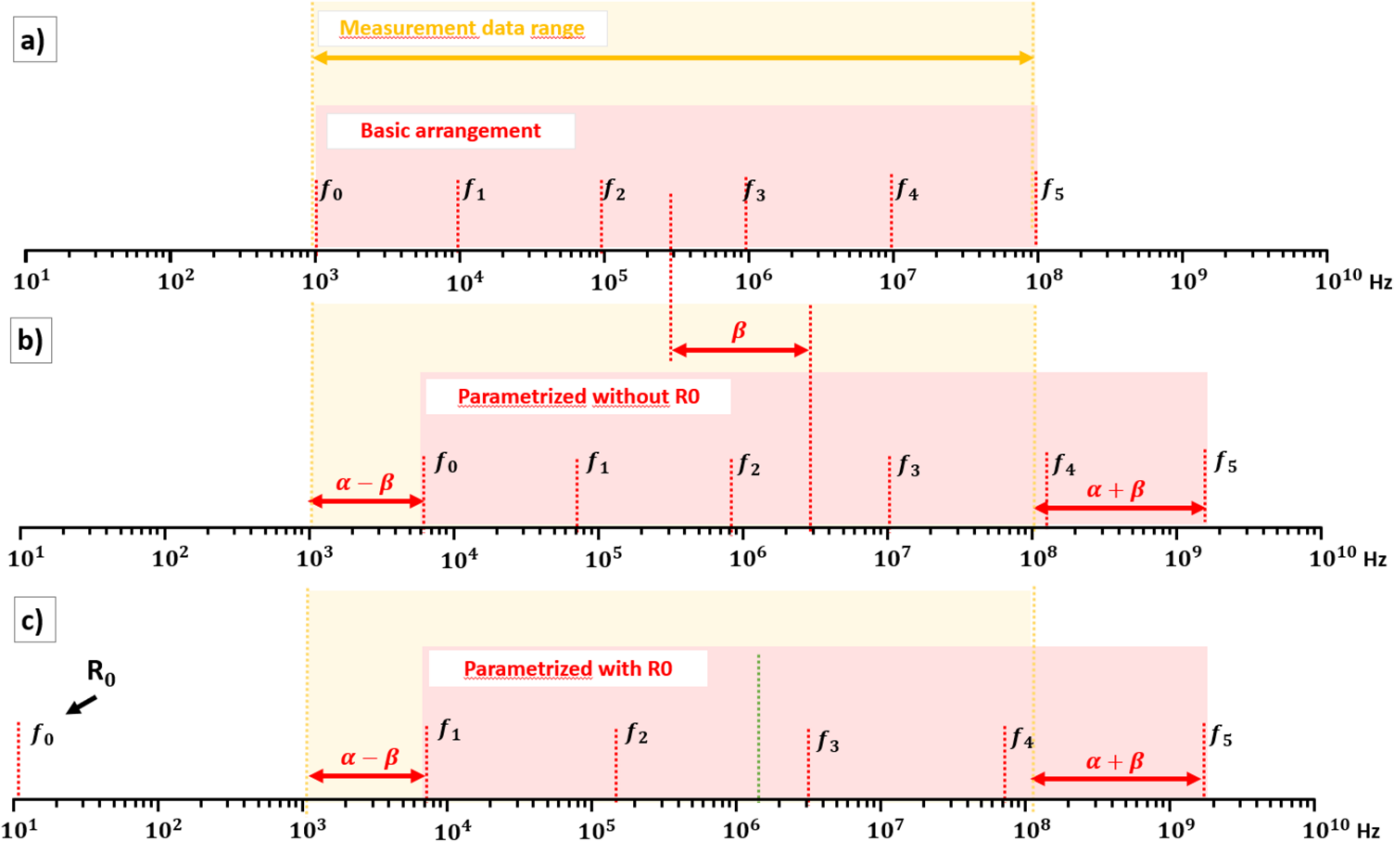
Schematic representation of characteristic frequencies $f_k$ of *LR* elements, marked as a red dashed line. The red region denotes the frequency range in which the frequencies $f_k$ are distributed. The yellow region denotes the measurement data range. The $\alpha$ and $\beta$ are parameters of $f_k$ distribution. Presented are 3 cases: (**a**) arrangement with uniform distribution in a logarithmic scale; (**b**) parameterized arrangement with the use of $\alpha$ and $\beta$; (**c**) parameterized arrangement with the use of $\alpha$ and $\beta$ and first frequency $f_0$ set far below measurement data range.

The distribution of characteristic frequencies that determine the properties of individual ladder elements, given by $f_k=R_k/(2 \pi L_k)$, is crucial for achieving a good agreement between the obtained model and the measurement data. The distribution of $f_k$ is arbitrarily selected and initially proposed as the simplest and trivial uniform distribution in a logarithmic scale within the frequency range of the measurement data (see Fig. 3a).

Next, a grid search was conducted for different distributions of $f_k$, parameterized by coefficients $\alpha$ and $\beta$ (see Fig. 3) to explore the possibility of finding a better solution than the baseline (uniform distribution).

A grid search method was performed using two parameters named $\alpha$ and $\beta$. The parameter $\alpha$ determines the number of decades by which the range of characteristic frequencies should be extended beyond the measurement range. A negative value of $\alpha$ narrows the range of characteristic frequencies (red area in Fig. 3) compared to the measurement data range. On the other hand, the parameter $\beta$ determines the number of decades by which the range of characteristic frequencies should be shifted to the right with respect to the measurement data range. A negative value of $\beta$ indicates a leftward shift. In order to improve the model fit and account for the presence of contact resistance in the measurements, which introduces a non-zero real resistance across the entire frequency range, a distribution with the first characteristic frequency set two decades below the measurement data (see Fig. 3c) was proposed. This effectively incorporates the real resistance $R_0$ component into the model in place of the first *RL* element.

## Results

### Basic results

**Figure 4:**
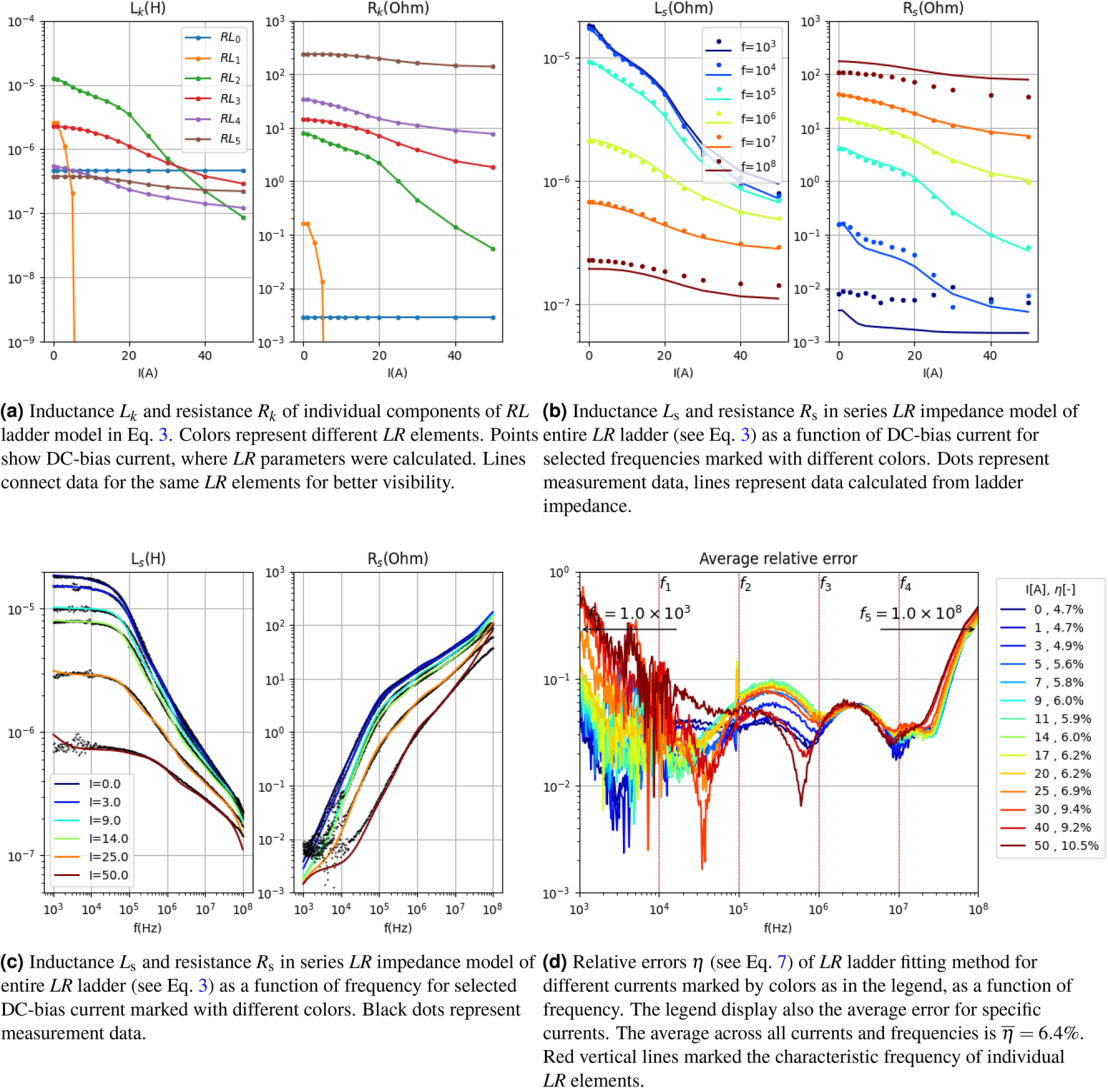
Presentation of circuit parameters of lumped elements model (Eq. 3) in the case of basic (uniform) arrangement (see Fig. 3a) of characteristic frequency $f_i$ for data presented in Fig. 2. Here $\overline{\eta }=6.4\%$.

The parameters of the default (basic) ladder configuration, shown in Fig. 4, represent the reference point for further improvements (see Fig. 3a). This solution can be considered as the baseline for subsequent refinements.

As a measure of the model quality, we utilized the relative error, denoted as $\eta =\eta (f,i)$, expressed by the following equation:

7 $$\begin{aligned} \eta =\eta (f,i)= \frac{|Z_\mathrm{model}(f,i) -Z_\mathrm{measur}(f,i) |}{Z_\mathrm{measur}(f,i)}. \end{aligned}$$

Moreover, we also employed the average $\eta$, denoted as $\overline{\eta }$, which represents the arithmetic mean across all values of *f* and *i*.

Figure 4d demonstrates that the global fitting, represented by $\overline{\eta }$, is below 6.5%, suggesting that it is sufficiently accurate for modeling purposes. The dependencies of $L_k$ (and consequently $R_k$) exhibit smooth variations with respect to current, ensuring good numerical stability. However, issues arise for the ladder element number $k=1$, where the value of $L_k$ remains below $10^{-9}$ H for most currents. In practical terms, this implies that this *LR* element does not contribute impedance to the entire ladder, rendering it effectively “unused”. Figure 4d illustrates that the largest error occurs at low frequencies, which may be attributed to the inclusion of the lead resistance and at high frequencies.

#### Parameterized uniform distribution

**Figure 5:**
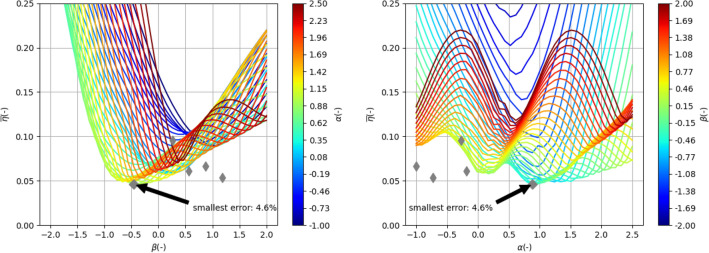
Average of relative error $\overline{\eta }$ across all currents and frequency for measurement data presented in Fig. 2 as a function of $\beta$ parameter for various $\alpha$ (left) and as a function of $\alpha$ parameter for various $\beta$ marked as color lines for characteristic arrangement without $R_0$ (see Fig. 3). A big gray diamond marks the best-found case. Additionally, best cases for other measurement data are marked with small diamonds.

The results of the grid search, considering two parameters, namely $\alpha$ and $\beta$, in terms of $\overline{\eta }$, are presented in Fig. 5. The best outcome was achieved at $\alpha =0.8$ and $\beta =-0.5$, yielding an $\overline{\eta }$ value of 4.6%. This represents an improvement of nearly 50% compared to the default distribution ($\alpha =\beta =0$). Furthermore, to assess the universality of the obtained $\alpha$ and $\beta$ parameters, a similar analysis was conducted using impedance measurement data from three additional rings, which were not presented in the Measurement Data section. It is evident that the optimal $\alpha$ and $\beta$, and consequently the initial pole distribution, vary depending on the measurement data. A more detailed quantitative comparative analysis is provided in the summary section.

**Figure 6:**
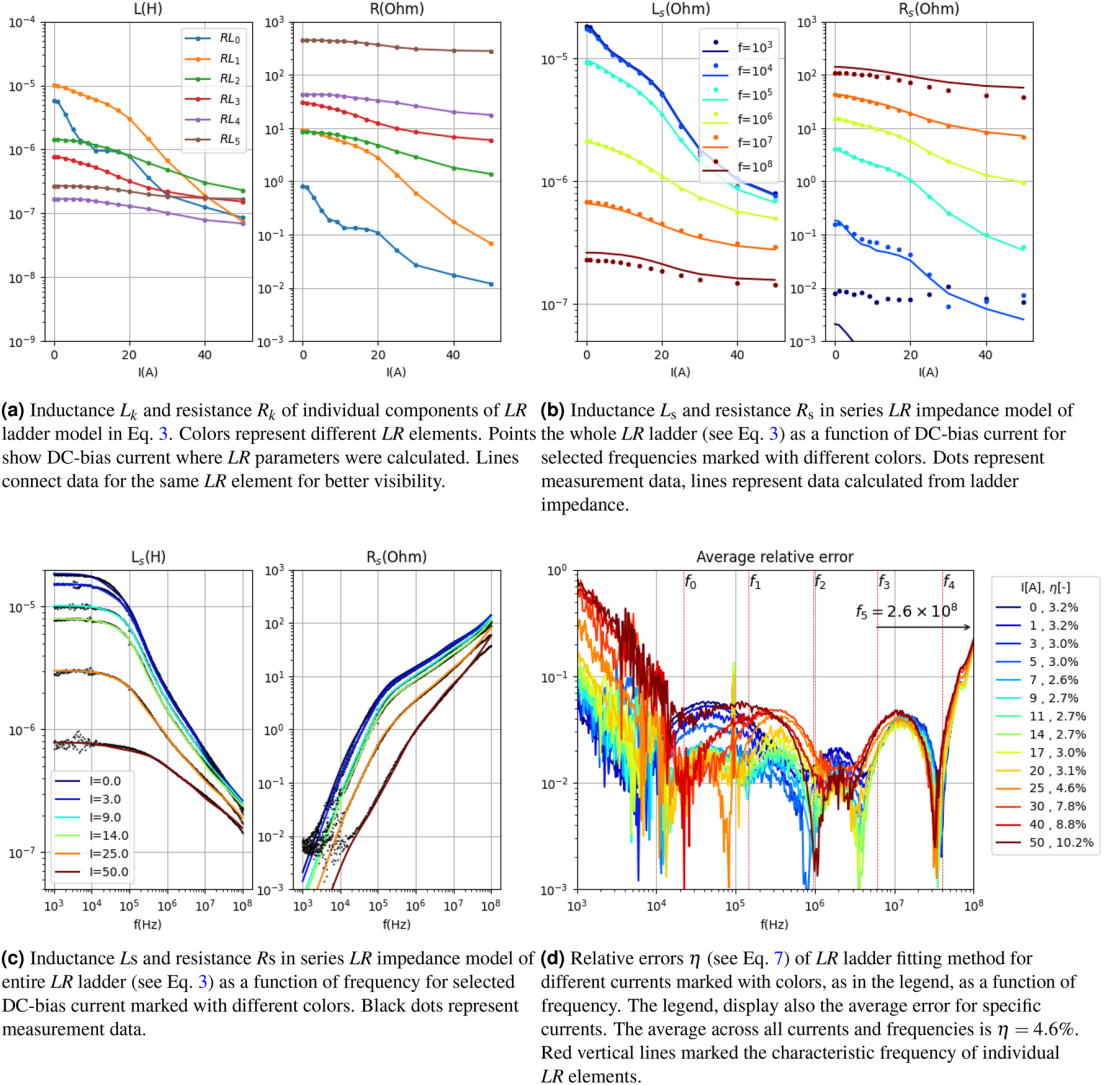
Presentation of circuit parameters of lumped elements model (Eq. 3) in the case of parametrized arrangement (see Fig. 3b) of characteristic frequencies $f_i$ for data presented in Fig. 2. Here $\overline{\eta }=4.6\%$.

The results for the best-case scenario (i.e., when $\overline{\eta }=4.6\%$) are presented in Fig. 6. Similar to the default distribution, both $L_k$ and $R_k$ exhibit continuous and smooth variations with respect to DC current (see Fig. 6a).However, a difference can be observed in the first ladder element, which has the lowest characteristic frequency ($f_0=2.1\times 10^4$ Hz). The dependency on current is not smooth in this case due to the presence of noise in the measurement data at the lowest frequencies. This can also be seen in the poor fitting of $\eta (f)$ at low frequencies (see Fig. 6d). The mismatch error for low frequencies primarily concerns the resistive component (see Fig. 6c). This arises from the fact that the fitted model does not exhibit the property of resistance converging to a constant value, as observed in the measurement data. To address this issue, a fixed resistive component, represented by a ladder element with a characteristic frequency well below the measurement range, is added to the model and described in the subsequent subsection.

#### Parameterized uniform distribution with $R_0$

**Figure 7:**
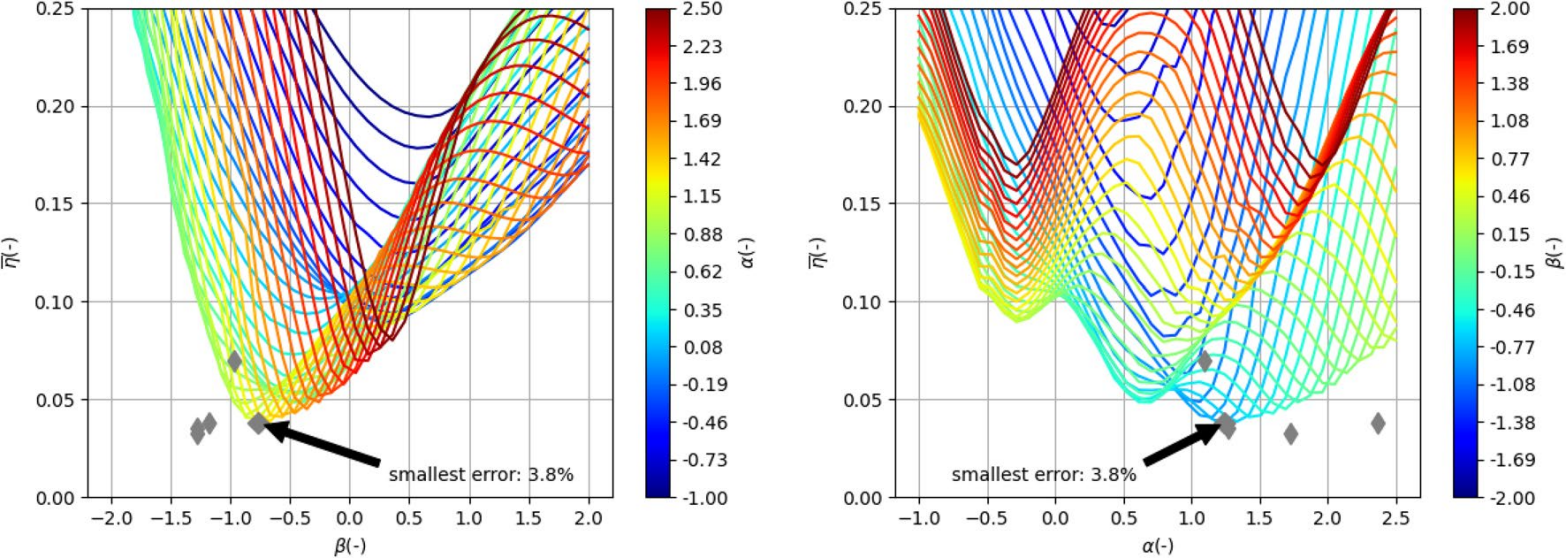
Average of relative error $\eta$ across all currents and frequency for measurement data presented in Fig. 2 as a function of $\beta$ parameter for various $\alpha$ (left) and as a function of $\alpha$ parameter for various $\beta$ marked with color lines for characteristic arrangement **with** $R_0$ (see Fig. 3) Big gray diamond marks best-found case. Additionally, best cases for other measurement data are marked with small diamonds.

In this section, we present the grid search results for a similar case as in the previous section, with the exception of imposing the position of the first characteristic frequency to be far (3 decades) below the lower frequency limit of the measurements. In the schematic diagram (Fig. 3), this corresponds to case (c). In such a scenario, within the frequency range of the measurements, only the resistive component is introduced, while the inductive component becomes very large for this *LR* element and can effectively be neglected (see Fig. 1a). Consequently, the first *LR* element effectively introduces only the parameter $R_0$. In the frequency range containing the measurements, a ladder with one less element was used, which theoretically could result in a larger error. The results of the fitted averages are presented in Fig. 7. It can be observed that the best case has a lower error compared to the system without $R_0$. Similarly, the measurement data for other rings (when compared to Fig. 5) also exhibit improved fitting. A more detailed comparative analysis is provided in the summary section.

**Figure 8:**
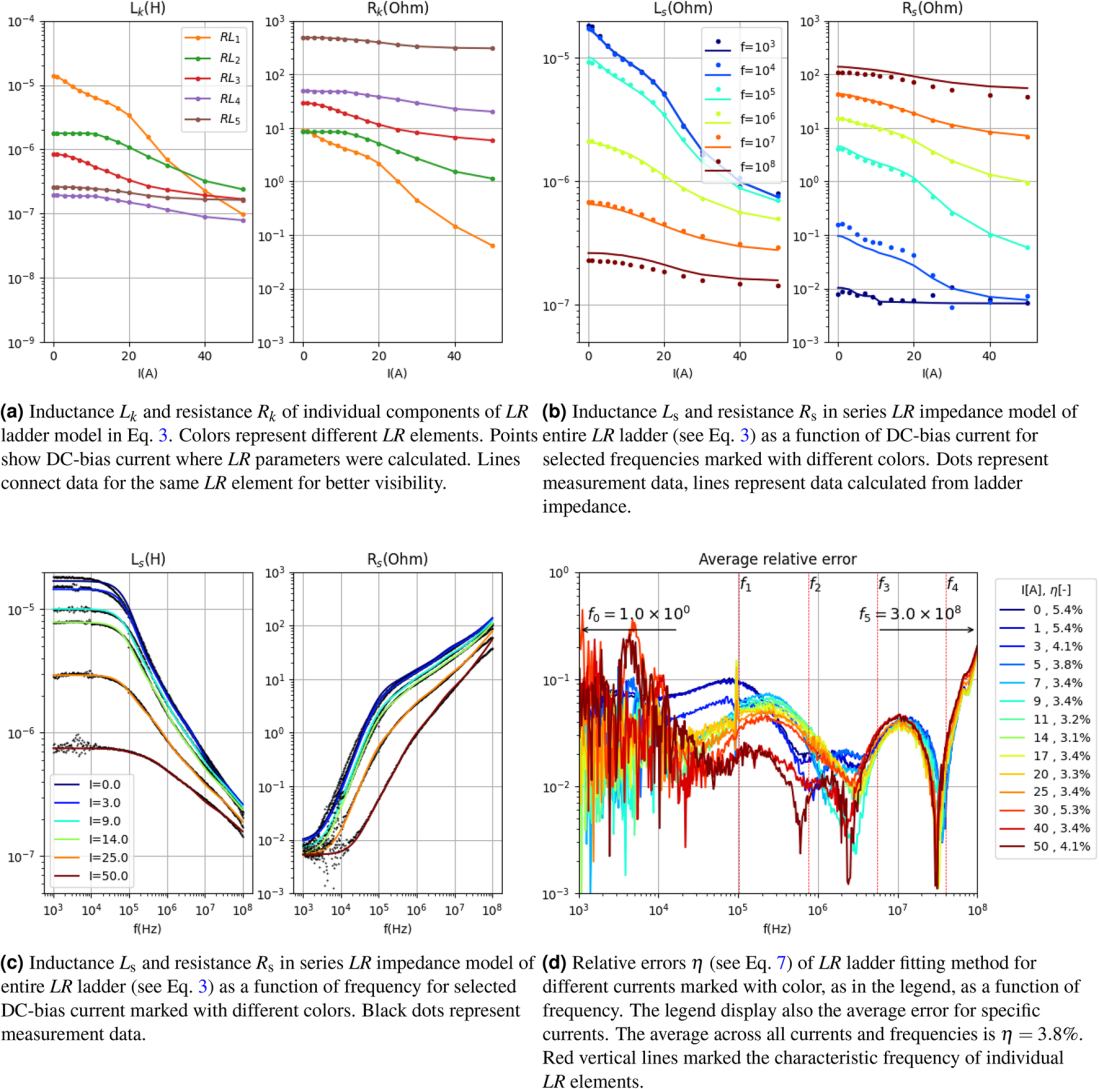
Presentation of circuit parameters of lumped elements model (Eq. 3) in the case of of parametrized arrangement (see Fig. 3c) of characteristic frequency $f_i$ for data presented in Fig. 2. Here $\overline{\eta }=3.8\%$.

The results for the best-case scenario (i.e., when $\overline{\eta }=3.8\%$) are presented in Fig. 8. These results correspond to the best model found for data presented in the measurement data section. It can be observed (see Fig. 8b,c) that, unlike in the previous section, the model accurately fits $R_\mathrm{s}$ in the low-frequency range. In Fig. 8b, where $R_\mathrm{s}$ is depicted, it is noticeable that at $f=10^3$ Hz, the resistance reaches a minimum value of $3\times 10^{-3}$ $\Omega$, which precisely corresponds to $R_0$. The shift in the characteristic frequency of the first ladder element has played its role. Figure 8d demonstrates that the error is below 4% in the majority of the frequency and current ranges. The largest errors occur in the low-frequency range, where the measurement data is most affected by noise.

### Summary

Achieving a good global fit requires finding the optimal distribution of characteristic frequencies for the ladder elements. The proposed approach of exploring the parameter space defined by $\alpha$ and $\beta$ yielded better fits compared to the basic approach of uniformly distributed characteristic frequency values in the frequency range of measurement data. Table 1 shows that the difference amounts to approximately 33%. Even better results were obtained by shifting one of the frequencies deep below the measurement range, which allowed for a better fit at low frequencies, taking into account the constant resistive component originating from lead resistances. In this case, the average results were improved by 54% compared to the basic (reference) case.

**Table 1:** Average relative error $\eta$(-) (Eq. 7) for the model fitted to measurement data series presented in Fig. 2 and four different magnetic ring measurement series not presented in this work.

Data type	Average$^1$ $\eta$(-)		
	Basic (%)	Without R$_0$ (%)	With R$_0$ (%)
This work	6.5	4.6	3.8
Ring 1	12.2	6.6	3.2
Ring 2	10.5	9.5	7.0
Ring 3	8.5	6.1	3.6
Ring 4	11.4	5.4	3.8

Rings 1–4 have similar $l_\mathrm{m}$ and $A_\mathrm{fe}$ as ring used in this work but different $\mu _r$.

${}^1$ Average is over all frequency and DC-bias current.

## Discussion

The presented results demonstrate that it is feasible to achieve a good fit for the nonlinear impedance model based on the *LR* ladder, with smoothly varying parameters *L* and *R* as a function of current with the use of classical methods. To achieve a good fit, finding the appropriate characteristic frequencies for each ladder element is necessary. For this purpose, a grid search analysis was conducted using two parameters ($\alpha$ and $\beta$), along with the shifting of one frequency far below the smallest frequency used in the measurements. The best solutions yielded results with an error approximately 50% smaller than the default assumption of evenly distributed characteristic frequencies within the measurement range. Such an obtained model can be utilized in circuit simulations. The basic model, although slightly inferior, may also prove to be useful.

This method can be further developed by exploring more precise frequency distribution methods, not necessarily based on uniform spacing. Additionally, it should be noted that the optimal distribution of frequencies depends on the excitation current, while here, the same distribution was used for all measurement data. Searching for the optimal distribution on a grid separately for each current will likely reduce the mean error, $\overline{\eta }$. However, special attention needs to be paid to the continuity of *L* and *R* as a function of current, as it is easier to maintain when using common characteristic frequencies for all currents. AI-based methods may well address the problem.

## Data Availability

The codes used to calculate the results of this study are available from the corresponding author upon reasonable request.
